# Peptidomes and Structures Illustrate Two Distinguishing Mechanisms of Alternating the Peptide Plasticity Caused by Swine MHC Class I Micropolymorphism

**DOI:** 10.3389/fimmu.2021.592447

**Published:** 2021-02-26

**Authors:** Xiaohui Wei, Song Wang, Zhuolin Li, Zibin Li, Zehui Qu, Suqiu Wang, Baohua Zou, Ruiying Liang, Chun Xia, Nianzhi Zhang

**Affiliations:** ^1^ Department of Microbiology and Immunology, College of Veterinary Medicine, China Agricultural University, Beijing, China; ^2^ Key Laboratory of Animal Epidemiology of the Ministry of Agriculture, China Agricultural University, Beijing, China

**Keywords:** swine MHC class I, micropolymorphism, RPLD–MS, immunopeptidome, crystal structure

## Abstract

The micropolymorphism of major histocompatibility complex class I (MHC-I) can greatly alter the plasticity of peptide presentation, but elucidating the underlying mechanism remains a challenge. Here we investigated the impact of the micropolymorphism on peptide presentation of swine MHC-I (termed swine leukocyte antigen class I, SLA-I) molecules *via* immunopeptidomes that were determined by our newly developed random peptide library combined with the mass spectrometry (MS) *de novo* sequencing method (termed RPLD–MS) and the corresponding crystal structures. The immunopeptidomes of SLA-1*04:01, SLA-1*13:01, and their mutants showed that mutations of residues 156 and 99 could expand and narrow the ranges of peptides presented by SLA-I molecules, respectively. R156A mutation of SLA-1*04:01 altered the charge properties and enlarged the volume size of pocket D, which eliminated the harsh restriction to accommodate the third (P3) anchor residue of the peptide and expanded the peptide binding scope. Compared with 99^Tyr^ of SLA-1*0401, 99^Phe^ of SLA-1*13:01 could not form a conservative hydrogen bond with the backbone of the P3 residues, leading to fewer changes in the pocket properties but a significant decrease in quantitative of immunopeptidomes. This absent force could be compensated by the salt bridge formed by P1-E and 170^Arg^. These data illustrate two distinguishing manners that show how micropolymorphism alters the peptide-binding plasticity of SLA-I alleles, verifying the sensitivity and accuracy of the RPLD-MS method for determining the peptide binding characteristics of MHC-I *in vitro* and helping to more accurately predict and identify MHC-I restricted epitopes.

## Introduction

The major histocompatibility complex (MHC) is a large genetic region that can encode a wide variety of molecules and consists of the highly polymorphic classical MHC class I (MHC-I) and MHC class II (MHC-II), which are central to the adaptive immune response (1). MHC-I is a cell surface glycoprotein that is responsible for the presentation of both endogenously and exogenously invading pathogen-derived peptide antigens for immune surveillance. The sequence diversity found in MHC-I ranges from micropolymorphisms, which contain just a few different amino acids, to differences of more than 30 amino acids in more distantly related allomorphs (2). This feature determines that the peptide-binding characteristics of individual MHC-I molecules are variable. Peptides anchor the antigen binding groove (ABG) *via* interactions between peptide residues and pockets, denoted A–F (3). The landscape of these pockets is usually determined by the composition of nearby amino acids. Polymorphisms alter the stereo- and electrochemical environment of the pockets, dictating their ability to accommodate different peptide residues, thereby influencing the nature and quantity of the bound peptides (4–6).

Besides MHC-I, the presented peptides on the cell surface also depend on the peptide loading complex in the endoplasmic reticulum (ER), which comprises chaperone proteins such as the transporter associated with antigen processing (TAP) and tapasin, among others. The cytoplasmic peptides are bound and transported into the ER lumen by TAP, and sometimes the peptide-binding preferences of TAP and human MHC-I (human leukocyte antigen class I, HLA-I) allotypes can strongly mismatch, indicating the existence of a TAP-independent compensatory peptide source (7–9). Tapasin has the ability to modulate peptide selection toward more stable ligands during peptide loading in the ER (10, 11). However, the high degree of polymorphism causes some HLA-I allotypes to have a stable peptide-free status and be able to efficiently assemble in tapasin-deficient cells (12, 13). Thus, the polymorphisms of MHC-I can shape the peptide repertoire by delineating the interactions with chaperones of the peptide-loading complex (7, 14).

At present, some studies have shown that humans carrying a polymorphism at a single amino acid in the ABG can shift the immune reactivity in many clinical scenarios (15–17). For instance, the polymorphism at residue 156 of human MHC-I (human leukocyte antigen class I, HLA-I) has been indicated to elicit divergent T cell immune reactivities (18, 19); B27 family members such as HLA-B*27:02, HLA-B*27:03, HLA-B*27:04, and HLA-B*27:05 confer a risk of ankylosing spondylitis, but HLA-B*27:06 and HLA-B*27:09 do not (20–22); the micropolymorphic allomorphs of the HLA-B35 family alter the presentation of identical ligands to affect immunogenicity and immunodominance hierarchies (23, 24); and distinct conformations of the same peptide presented by HLA-B7 family alleles have been reported to favor distinct escape mutations in human immunodeficiency virus (HIV) (25). In addition to affecting the presentation of peptides, polymorphisms can also affect the binding of HLA-I molecules to inhibitory or activating receptors on different immune cells, thereby affecting the adaptive or natural immune response (18, 19, 26). These correlations between polymorphisms of the MHC-I molecule, and diseases are still a challenge to fully and accurately explain its mechanism.

Recently, the abundant usage and improvement of high-quality mass spectrometry (MS) to determine eluted HLA-I peptidome data provided a new solution to this problem (27, 28). For example, Abelin JG et al. established a strategy to identify the peptide-binding motif of mono HLA-I using CRISPR gene edited cell lines (29), and the influences of the HLA-B57 micropolymorphism on the immunopeptidome were defined by this method (2). These new MS methods were powerful in the identification of the immunopeptidomes of HLA-I alleles with complete research conditions, but it is still difficult to study the various animal MHC-I alleles lacking suitable cell lines and antibodies, such as swine MHC-I molecules (termed swine leukocyte antigen class I, SLA-I). We showed that the arginine at position 156 (156^Arg^) is essential for peptide binding of SLA-1*04:01, and when 156^Arg^ was mutated to alanine, the peptides bound by the mutant SLA-1*04:01 (R156A) became significantly different from those bound by SLA-1*04:01 (30). Another previous study reported that SLA-1*13:01 is quite similar to SLA-1*04:01 but has different properties in presenting peptides (31). The lack of corresponding cell lines and monoclonal antibodies against SLA-I makes it challenging for us to use the current MS method to comprehensively and accurately determine their differences in peptide binding caused by micropolymorphism. Considering the time cost and financial budget, it is unrealistic to establish necessary experimental conditions for many animal MHC-I molecules. We need a more rapid and economical method to determine the peptide binding motif of an animal MHC-I molecule.

The *de novo* peptide sequencing technique for data-independent acquisition MS has been used to identify immunopeptidomes that do not exist in any database (27, 32, 33). We developed a new *in vitro* method to identify the immunopeptidome of MHC-I by a random peptide library combined with LC-MS/MS and *de novo* sequencing. The random peptide library was refolded with MHC-I heavy chain and beta-2-microglobulin (*β*2-M) to form the peptide-MHC-I complex (pMHC-I). Then, pMHC-I was purified, and the bound peptides were eluted and sequenced by LC-MS/MS and *de novo* sequencing. Ultimately, the peptide-binding motif of MHC-I was determined. Using this method, the peptide binding properties of MHC-I molecules of two species, bat and frog, were identified without the limitations of cell lines and antibodies (34, 35).

We believe that this new method of combining random peptide library refolding and MS *de novo* sequencing is also helpful to solve the problem of how a micropolymorphism affects the peptide-binding plasticity of animal MHC-I molecules, because this method can rapidly and economically determine the peptidomes bound by MHC-I mutants and similar allotypes. Here we utilized this newly developed method to map unbiased micropolymorphism-dependent immunopeptidome changes in SLA-I, SLA-1*04:01, mutant SLA-1*04:01(R156A), and a similar allotype SLA-1*13:01. The key residues, residues at positions 99 and 156 (residues 99 and 156), adopted two distinguishing manners to alter the peptide binding plasticity of SLA-I molecules, and their molecular mechanisms were further demonstrated by the corresponding crystal structures. This study contributes to understanding the link between MHC-I micropolymorphisms and peptide-presenting plasticity and helped us to more accurately predict and identify SLA-I restricted epitopes.

## Materials and Methods

### Synthesis of Epitopes and Random Peptide Repertoire

All potential virus-derived epitopes binding to SLA-1 were predicted by the NetMHCpan 4.0 server (http://www.cbs.dtu.dk/services/NetMHCpan/) and synthesized by the method of solid phase peptide synthesis (SPPS) (**Table 1**). These peptides were then purified to 99% by reverse-phase high-performance liquid chromatography (RP-HPLC) and MS (SciLight Biotechnology). A random peptide repertoire was synthesized in which every position was distributed at an equal molar ratio with 19 amino acids other than cysteine. The distribution of amino acids in each position of the random peptide repertoire was verified by liquid chromatography-tandem mass spectrometry (LC-MS/MS) and *de novo* sequencing (32, 33). These peptides were stored in lyophilized aliquots at −80°C after synthesis and dissolved in dimethyl sulfoxide (DMSO) before use.

**Table 1:** Virus-derived epitopes predicted and evaluated to bind SLA-1 by *in vitro* refolding.

Name	Derived protein	Sequence	pSLA-1*13:01^a^	pSLA-1*04:01^a^	pSLA-1*04:01(R156A)^a^
S-OIV_NW9_	Influenza-NA	NSDTVGWSW	–	+	+
S-OIV_GY9_	Influenza-PA	GTFDLGGLY	–	−	+
S-OIV_WY9_	Influenza-PB2	WSQDPTMLY	–	−	+
S-OIV_YY9_	Influenza-HA	YVFVGSSRY	–	−	+
PRRSV_AY9_	PRRSV-GP2	ASDWFAPRY	–	+	+
PRRSV_GF9_	PRRSV-GP5	GTDWLAQKF	–	+	+
FMDV_MY9_	FMDV-polyprotein	MTAHITVPY	–	+	+

a The ability of epitopes to bind SLA-1*13:01/SLA-1*04:01/SLA-1*04:01(R156A). “+” indicates the corresponding complex is stable and can tolerate anion-exchange chromatography; “−” indicates the peptides cannot stabilize the complexes.

### Protein Preparation

DNA fragments encoding extracellular domains of SLA-1*04:01 (GenBank accession No. EU170457.1, residues 1–275 of the mature protein), SLA-1*13:01 (GenBank accession No. AB847437.1, residues 1–275 of the mature protein), and swine *β*2-M (s*β*2-M, GenBank accession No. BAG32341, residues 1–98 of the mature protein) were cloned *via* reverse transcription (RT)-PCR (TransGen Biotech) with allele group-specific PCR primer pairs designed for SLA-1 alleles using total RNA extracts isolated from the kidneys of Landrace swine. The mutant SLA-1 was cloned by overlap PCR. Then, the gene fragments were cloned into pET-21a (+) vectors (Novagen) and expressed as inclusion bodies in *E. coli* BL21 (DE3). The recombinant proteins were purified as described previously and dissolved in 6 M guanidine hydrochloride (Gua-HCl).

### Assembly of the pSLA-1 Complexes

The proteins were refolded by the gradual dilution method using buffer containing 400 mM L-Arg HCl, 2 mM EDTA, 5 mM GSH, 0.5 mM GSSH, and 100 mM Tris-HCl (pH 8) at 277 K for 12 h. The SLA-1 and s*β*2-M inclusion bodies were individually added to the refolding buffer containing peptides or the random peptide repertoire at a 1:1:5 molar ratio. The refolded complexes were concentrated and purified with a Superdex 200 16/60 column in Tris buffer (20 mM Tris [pH 8] and 50 mM NaCl), followed by Resource Q anion-exchange chromatography (GE Healthcare) in Tris buffers (Buffer A: 20 mM Tris [pH 8] and 5 mM NaCl; Buffer B:20 mM Tris [pH 8] and 500 mM NaCl).

### Isolation of the High-Affinity Peptide Repertoire and LC-MS/MS Sequencing

The SLA-1*04:01, SLA-1*13:01 and their mutants were renatured with s*β*2-M and random nonapeptide repertoire in the same manner. Two replicates of each allele are assessed simultaneously. The complexes were formed by refolding and then purified by gel filtration and anion-exchange chromatography as described above. The peptide-containing fractions were eluted by acidification with 10% acetic acid and incubated at 65°C for 15 min. Next, the peptides were concentrated using a 3-kDa filter and desalted using C18 tips as described in previous reports (34). In brief, the desalting tips were first revitalized using 200 μl of methanol and then equilibrated with 200 μl of 0.1% (v/v) trifluoroacetic acid (TFA). Second, the peptides were washed twice with 200 μl of 0.1% (v/v) TFA and eluted with 200 μl of a solution containing 0.1% (v/v) TFA and 75% (v/v) acetonitrile.

The desalted peptides were separated using the EasyNano LC 1000 system (Thermo Fisher Scientific, San Jose, California). The peptide components were loaded into a trap column (5-μm pore size, 150-μm inner diameter [i.d.], ×3-cm length, 100 Å) and separated by a custom-made C18 column (3-μm pore size, 75-μm i.d., 315-cm length, 100 Å) with a flow rate of 450 μl/min. A 60-min linear gradient was applied as follows: 3% B (0.1% formic acid in acetonitrile [v/v]/97% A (0.1% formic acid in H_2_O [v/v]) to 6% B in 8 min, 6% B to 22% B in 37 min, 22% B to 35% B in 8 min, 35% B to 100% B in 2 min, and 100% B for 5 min. The acquisition of MS data was performed using a Q Exactive HF (Thermo Fisher Scientific, Bremen) in the data-dependent acquisition mode. The top 20 precursors by intensity from the mass range of m/z 300 to 1800 were sequentially fragmented with the higher energy collisional dissociation and normalized collision energy 27. The dynamic exclusion time was 20 s. The automatic gain control for MS1 and MS2 was set to 3e6 and 1e, and the resolution for MS1 and MS2 was set to 120 and 30K, respectively.

### *De Novo* Analysis and Peptide Scoring

Based on the LC-MS/MS spectrum information, the Peaks Studio software resolved each of the peptides from each spectrum (false discovery rate = 1%), as previously reported (34). The parameters were set as follows: the enzyme was set to no specific, the variable modifications were adjusted oxidation (M)/deamidated (N,Q), the peptide mass tolerance was approximately ±10 ppm, and the fragment mass tolerance was set to 0.02 Da. The score was based on the similarity between each spectrum that matched the peptide and the theoretical fragmentation peak of the peptide. The identified peptides were adjusted by the detection threshold (length = 9, score ≥50) as previously reported (34, 36, 37). First, the restricted motif of presentation needed to be determined by calculating the standard deviation (SD, *σ*) and average value $\left(\bar{X}\right)$, as well as the coefficient of variation (*V s*) as follows (38):

$$V\;s=\frac{\sigma }{\bar{X}}$$

The likelihood of each amino acid at every position of the peptides was computed. We assumed that Ile made up half of Leu. The position with a higher *V s* was considered to be a restricted position in the presentation. Based on the weighting probability (the position-probability matrix) of every amino acid located at a single locus of nine (39, 40), the specific amino acids and elements at the restricted motifs (the number of restricted motifs *N_res_*) were valued (*M_k,j_*) as follows:

$$M_{k,j}=\frac{1}{N}\sum_{i=1}^{N}I\left(X_{i,j}-k\right)$$

where *N* is the number of peptides identified from *de novo* sequencing, and *X_i,j_* belongs to the set of alphabet symbols, given *i*ϵ (1, …, *N*) and *j*ϵ (1, …, *N_res_*), which represent the amino acid species with abbreviations at specific positions. *k* is the set of alphabet symbols, and *I*(*X_i,j_* = *k*) is an indicator function where *I*(*X_i,j_* = *k*) is valued as 1 when *X_i,j_* = *k* and 0 otherwise. After the amino acid type at a specific position was valued, the target peptides were then scored (*S*) through the added values of the specific amino acids at their restricted motifs (specific *M_k,j_* is represented as *δ_j_*) as follows:

$$S=\sum_{j=1}^{N_{res}}\delta_{j}$$

The peptide illustrations were drawn using WebLogo (http://weblogo.berkeley.edu/logo.cgi) (41). The height of the stack indicates the sequence conservation at that position, while the height of symbols within the stack indicates the relative frequency of each amino acid at that position. The heat maps between alleles were drawn by Icelogo (https://iomics.ugent.be/icelogoserver/logo.html) (42). For each position, the amino acid frequencies in the positive set were compared with the reference set. At the bottom of the heat map, the gradient shows which *P*-value correlates with which color. According to the given *P*-value = 0.05, only the significantly up-regulated and down-regulated elements are colored in green and red, respectively. The *Z*-score was used to calculate the position and amino acid-specific *P*-value and was calculated using the following formula:

$$Z-score=\frac{X-\mu }{\sigma }$$

The formula calculates how many times the frequency (*X*) of the amino acid at the position deviates from the average value (*μ*, the frequency of a specific amino acid at a position in the reference set) based on the calculated standard deviation (σ). An error funssction can calculate a *P*-value for this *Z*-score.

$$P-value=erf\left(\frac{Z-score}{\sqrt{2}}\right)$$

### Thermal Stabilities of the pSLA-1 Molecules

The circular dichroism (CD) spectra of the peptide-SLA-1 (pSLA-1) complexes were obtained on a CD instrument (Chirascan; Applied Photophysic, Ltd.). The CD spectrum was measured using a Jasco J-810 spectrometer equipped with a water-circulating cell holder. A 1-mm optical path length cell was used for monitoring at 218 nm as the temperature increased from 20 to 80°C at a rate of 1°C/min. The temperature of the solution was detected using a thermistor. The ratio of unfolded protein to the mean residue ellipticity (*θ*) was calculated. The unfolded fraction is shown as (*θ − θ_N_)*/(*θ_U_ − θ_N_*), where *θ_N_* and *θ_U_* are the mean residue ellipticity values in the fully folded and the fully unfolded states, respectively. The midpoint transition temperature (*Tm*) was computed by denaturation curve data in the Origin 9.1 program (OriginLab).

### Crystallization and Data Collection

Crystallization experiments were performed using the sitting-drop and hanging-drop vapor diffusion methods at 277 K and 291 K, respectively. The crystal of SLA-1*04:01 with the peptide MTAHITVPY derived from FMDV (MY9) (pSLA-1*04:01_MY9_) was obtained with Index solution No. 54 (0.05 M calcium chloride dihydrate, 0.1 M Bis-Tris (pH 6.5), and 30% v/v polyethylene glycol (PEG) monomethyl ether 550). The pSLA-1*04:01 (R156A)_MY9_ crystal was obtained with PEG/ion solution No. 6 (0.2 M sodium chloride and 20% w/v PEG 3350). The crystal of SLA-1*13:01 with mutated peptide ESDTVGWSW (EW9) (pSLA-1*13:01_EW9_) crystal was obtained with Index solution No. 96 (0.15 M potassium bromide and 30% w/v PEG monomethyl ether 2000). The crystal of SLA-1*13:01 with the peptide mutant NSDTVGWSW (NW9) (pSLA-1*13:01 (F99Y)_NW9_) was obtained with Index solution No. 46 (0.1 M Bis-Tris pH 6.5 and 20% w/v PEG monomethyl ether 5000). Prior to X-ray diffraction, the crystals were soaked for several seconds in reservoir solution containing 17% glycerol as a cryoprotectant and then flash-cooled in a stream of gaseous nitrogen at 100 K. Diffraction data for pSLA-1*04:01_MY9_ and pSLA-1*04:01 (R156A)_MY9_ were collected to resolutions of 2.0 Å and 1.8 Å, respectively, at Beamline BL17U (wavelength, 0.97892 Å) of the Shanghai Synchrotron Radiation Facility (Shanghai, China) using an R-AXIS IV++ imaging plate detector. Diffraction data for pSLA-1*13:01_EW9_ and pSLA-1*13:01 (F99Y)_NW9_ were collected to resolutions of 1.8 Å and 2.4 Å, respectively, at Beamline BL18U1 (wavelength, 0.97776 Å). The data were autoindexed, integrated, scaled, and merged using the HKL-3000 software package (HKL Research) (43). The crystallographic statistics for the complexes are listed in **Table 2**.

**Table 2:** X-ray diffraction data processing and refinement statistics.

Parameter	SLA-1*13:01_EW9_	SLA-1*13:01 (Y99F)_NW9_	SLA-1*04:01_MY9_	SLA-1*04:01 (R156A) _MY9_
Data collection				
Space group	C121	C121	C121	P12_1_1
Unit cell parameters (Å)	218.57, 45.78, 45.45 90.00, 90.276, 90.00	218.31, 46.31, 45.02 90.00, 90.65, 90.00	88.67, 77.15, 62.32 90.00, 119.42, 90.00	49.04, 66.20, 57.03 90.00, 107.91, 90.00
Resolution range (Å)	53.93–1.80 (1.85–1.80)	50.00–2.37 (2.41–2.37)	50.00-2.00 (2.07–2.20)	50.00–1.80 (1.86–1.80)
Total reflections	273,309	100,759	145,127	111,577
Unique reflections	38,597	17,844	23,996	29,750
*R*_merge_ (%)*^b^*	11.7 (23.4)	15.9 (24.1)	5.7 (15.7)	6.4 (13.7)
Avg *I*/σ(*I*)	9.9 (2.2)	8.4 (5.7)	26.535 (12.486)	18.924 (11.338)
Completeness (%)	93.8	98.3	96.8	97.1
Redundancy	7.1 (7.4)	5.6 (5.5)	6 (6.2)	3.8 (4.0)
Refinement				
Resolution(Å)	53.928–1.803	34.536–2.400	28.58-2.500	35.712-1.798
No. of reflections	38,582	15,565	11,972	29,741
*R*_factor_ (%)*^c^*	20.40	22.21	23.8	19.33
*R*_free_ (%)	22.31	26.44	28.8	24.47
R M S. deviations				
Bonds (Å)	0.004	0.006	0.005	0.008
Angles (°)	0.765	0.785	0.809	0.950
Average B factor	35.789	41.925	43.08	20.08
Ramachandran plot quality				
Most favored region (%)	97.85	95.2	97.35	96.28
Allowed region (%)	2.15	4.80	2.65	3.72
Disallowed region (%)	0.00	0.00	0.00	0.00

a Values in parentheses are for the highest-resolution shell.

b Rmerge = Σ_hkl_Σ_i_|I_i_(hkl) − 〈I(hkl)〉|/Σ_hkl_Σ_i_ I_i_(hkl), where I_i_(hkl) is the observed intensity, and 〈I(hkl)〉is the average intensity from multiple measurements.

c R = Σ_hkl_|| F_obs_ | − k | Fcalc | |Σ_hkl_| F_obs_|, where R_free_ is calculated for a randomly chosen 5% of reflections, and R_work_ is calculated for the remaining 95% of reflections used for structure refinement.

### Structural Determination and Analysis

The structures of all SLA complexes were determined by molecular replacement with the Phaser program using SLA-1*04:01 (Protein Data Bank (PDB) ID: 3QQ3, with the peptide excluded) as a search model (44). The comprehensive model was rebuilt manually using COOT (45), and refinement was restrained with REFMAC5 (46). Refinement rounds were implemented using the phenix.refine program in the PHENIX package with isotropic atomic displacement parameter (ADP) refinement and bulk solvent modeling (47). Finally, the PROCHECK program was used to assess the stereochemical quality of the final model (48). The structural illustrations and the electron density-related figures were drawn using PyMOL (https://www.pymol.org). Multiple-sequence alignment was performed with Clustal Omega (http://www.ebi.ac.uk/Tools/msa/clustalo/). The accessible surface area (ASA) and buried surface area (BSA) were calculated with PDB in Europe Proteins, Interfaces, Structures and Assemblies (PDBePISA, http://www.ebi.ac.uk/msd-srv/prot_int/pistart.html), and the B factor was calculated with CCP4.

### Data Deposition

The coordinates and structural factors of the SLA complex have been deposited in the Protein Data Bank (http://www.rcsb.org/pdb/home/home.do) under the following accession numbers: pSLA-1*13:01EW9, 6KWO; pSLA-1*13:01(F99Y)NW9, 6KWN; pSLA-1*04:01MY9, 6KWK; pSLA-1*04:01(R156A)MY9, 6KWL.

The mass spectrometry proteomics data have been deposited in the ProteomeXchange Consortium via the PRIDE (https://www.ebi.ac.uk/pride/) partner repository with the dataset identifier PXD019523.

## Results

### Residues 99 and 156 Could Impact the Peptide Binding of SLA-1*13:01 and SLA-1*04:01

**Figure 1:**
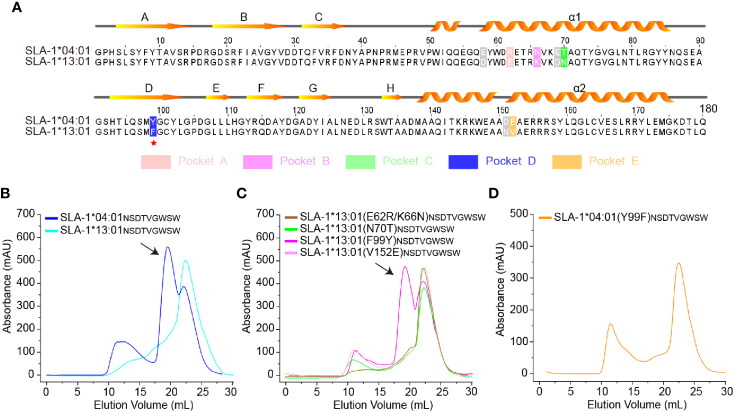
Structure-based alignment and *in vitro* refolding comparison between SLA-1*04:01 and SLA-1*13:01. **(A)** Structure-based sequence alignment of SLA-1*04:01 and SLA-1*13:01. Orange arrows above the alignment indicate β-stands; cylinders denote α-helices. Differential residues in pockets are highlighted in different colors; gray residues indicate that they are out of the pockets. **(B–D)** Mutagenesis and *vitro* refolding experiments. Gel filtration chromatograms of the refolded products obtained using a Superdex 200 10/300 GL column (GE Healthcare). The black arrows point to the peak of the compound. **(B)** The gel filtration chromatograms of the *in vitro* refolding test of SLA-1*04:01 and SLA-1*13:01 with the NW9 peptide. **(C)** Mutagenesis and *in vitro* refolding experiment of SLA-1*13:01 with the NW9 peptide. **(D)** The *in vitro* refolding experiment of mutant SLA-1*04:01 (Y99F).

The classic SLA-I allele, SLA-1*13:01, has up to 97% homology with SLA-1*04:01, but they had very different properties in presenting peptides (**Table 1**), which is consistent with previous reports (31). There are only five different residues (positions 62, 66, 70, 99, and 152) in the pockets (**Figure 1A**), but SLA-1*13:01 could not bind to most of the SLA-1*04:01 restricted peptides (**Table 1** and **Figure 1B**). Via *in vitro* point mutations and refolding experiments, different mutations at residue 99 were verified as the key residues (**Figures 1B, C**). Only SLA-1*13:01 (F99Y) could bind to peptide NSDTVGWSW (NW9 for short), which was crystallized with SLA-1*04:01 previously (30). The reverse mutation of SLA-1*04:01 (Y99F) confirmed that 99^Tyr^ was critical because it could not bind peptide NW9 (**Figure 1D**).

Our previous studies have also shown that residue 156 is critical for SLA-1*04:01 antigen binding (30). Based on previous reports, the residue at position 156 (residue 156) is the potential key site associated with peptide presentation of HLA-I and T-cell receptor (TCR) specificity (18). The peptide-binding results showed that the SLA-1*04:01 (R156A) mutant was able to bind more peptides than SLA-1*04:01 **(** **Table 1**).

### Immunopeptidomes Showed the 99^Tyr/Phe^ Micropolymorphism Could Narrow the Range of Peptide Binding of SLA-1*13:01 Compared With SLA-1*04:01 by Affecting the P1 Residues

**Figure 2:**
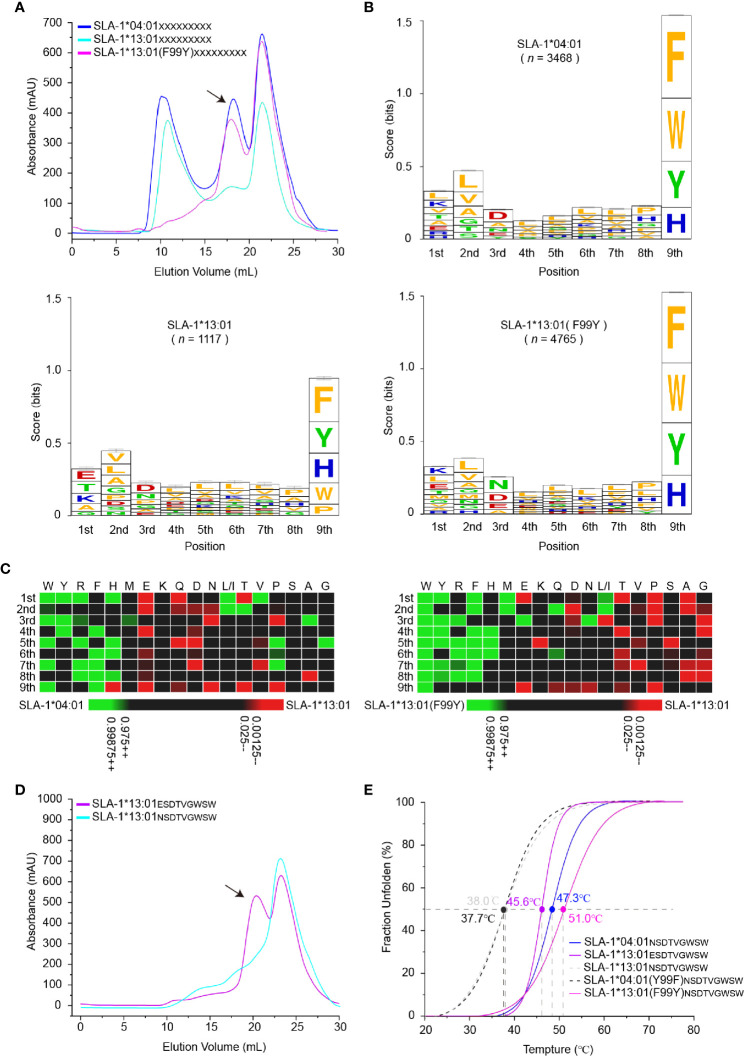
Determination of variation-dependent changes in residue 99 between SLA-1*04:01 and SLA-1*13:01. **(A)** The visual display of SLA-1*04:01, SLA-1*13:01, and its mutant *in vitro* refolding efficiency with the random nonapeptide repertoire by gel filtration chromatograms. The black arrows point to the peak of the compound. **(B)** Visual analysis of reliable peptides identified from LC-MS/MS and *de novo* sequencing by the WebLogo website (http://weblogo.berkeley.edu/). Amino acids are represented by the single-letter code with the height scaled to prevalence and color representing basic (blue), acidic (red), polar (green), and hydrophobic (orange) residues. Only amino acids with a 5% or greater prevalence are depicted. *n* is the number of peptides within the data set. Each column of amino acids has an error bar at the top. The height of the y-axis is the maximum entropy for the given sequence type (log_2_20 _=_ 4.3 bits). **(C)** Comparison of motifs between alleles *via* heatmap analysis from the IceLogo website (https://iomics.ugent.be/icelogoserver/). The color (green or red) indicates a significant difference (*P* < 0.05) in the amino acid at the position between two allele motifs. **(D)** Measure of SLA-1*13:01 refolding efficiency with mutated peptide EW9. The black arrows point to the peak of the compound. **(E)** Thermal stabilities of pSLA-1 complexes analyzed by the CD spectrum. The stabilities can be measured by the *Tm* value. The *Tm* values of the complexes are labeled.

To map the micropolymorphism 99-dependent changes in the peptide binding, the immunopeptidomes of SLA-1*04:01, SLA-1*13:01 and its mutant SLA-1*13:01 (F99Y) were determined using RPLD-MS. The *in vitro* refolding peak of SLA-1*13:01 was much lower than that of SLA-1*04:01 and SLA-1*13:01 (F99Y) (**Figure 2A**). The numbers of peptidomes of SLA-1*04:01 (*n* = 3,468) and SLA-1*13:01 (F99Y) (*n* = 4,765) were significantly different from that of SLA-1*13:01 (*n* = 1,117) (**Table S1**). This finding implied that residue 99 greatly affected the number of binding peptides.

Our previous studies have shown that P2/P3 and P*Ω* residues are calculated as the main anchor points of SLA-I restricted peptides, inserting into the B/D and F pocket, respectively (30, 49, 50). The nonapeptide binding motif of SLA-1*04:01 determined by RPLD-MS perfectly matched the existing conclusion derived from structural and peptide binding data: P*Ω* has obvious selectivity with aromatic amino acids; the B pocket allows many hydrophobic and small residue insertions; and the D pocket prefers the negatively charged or small amino acids (30). The high consistency of the peptide binding motifs of SLA-1*04:01 determined by the two different methods demonstrated the credibility of our new method.

Although the frequencies of amino acids appearing at the P2 and P3 positions were not exactly the same, the preferences of these major anchor residues were not significantly changed by the F99Y mutation (**Figures 2B, C**). Comparing the preferences of all positions, the only significant change occurred at the P1 residue among SLA-1*13:01, SLA-1*13:01 (F99Y), and SLA-1*04:01. The negatively charged residue Glu was on top of SLA-1*13:01, but the positively charged residue Lys was the first residue of SLA-1*13:01 (F99Y) and SLA-1*04:01 (Ile and Leu both contributed to the amount of “L”, so we assumed that the ratio of Ile and Leu was equal to 50%) (**Figure 2B**). In general, the A pocket accommodates the P1 residue and interacts with the carbon backbone of the P1 residue through the conserved amino acids in the pocket. It has now been found that the P1 residue can affect peptide binding only in rare cases (51).

To explore whether the 99^Tyr/Phe^ micropolymorphism influenced the P1 residue, we created a mutant peptide ESDTVGWSW (EW9 for short), which was only P1-Glu different from the NW9 peptide, to test its binding property to SLA-1*13:01. The mutant peptide EW9 could easily renature SLA-1*13:01 as expected (**Figure 2D**). The stabilities of these pSLA-I complexes were measured *via* the circular dichroism (CD) spectrum, and the midpoint transition temperature (*Tm*) values of SLA-1*13:01_NW9_ and SLA-1*04:01 (F99Y)_NW9_ were lower than those of SLA-1*13:01 (F99Y)_NW9_ and SLA-1*04:01_NW9_ (**Figure 2E**). For the EW9 peptide, the *Tm* value exceeded the 99^Phe^ unstable complexes, but it was less than the *Tm* values of the 99^Tyr^ compounds (**Figure 2E**). This result indicated that P1-Glu could form some bonds with the A pocket to stabilize pSLA-1*13:01 even if the force was not sufficiently strong compared with the force formed by 99^Tyr^ with peptides. In addition, micropolymorphism 99 could greatly affect the peptide binding motif and the affinity of SLA-I by altering the P1 anchor residue.

### The Effect of the 99^Tyr/Phe^ Micropolymorphism on Peptide Binding, as Illustrated by the Crystal Structures

**Figure 3:**
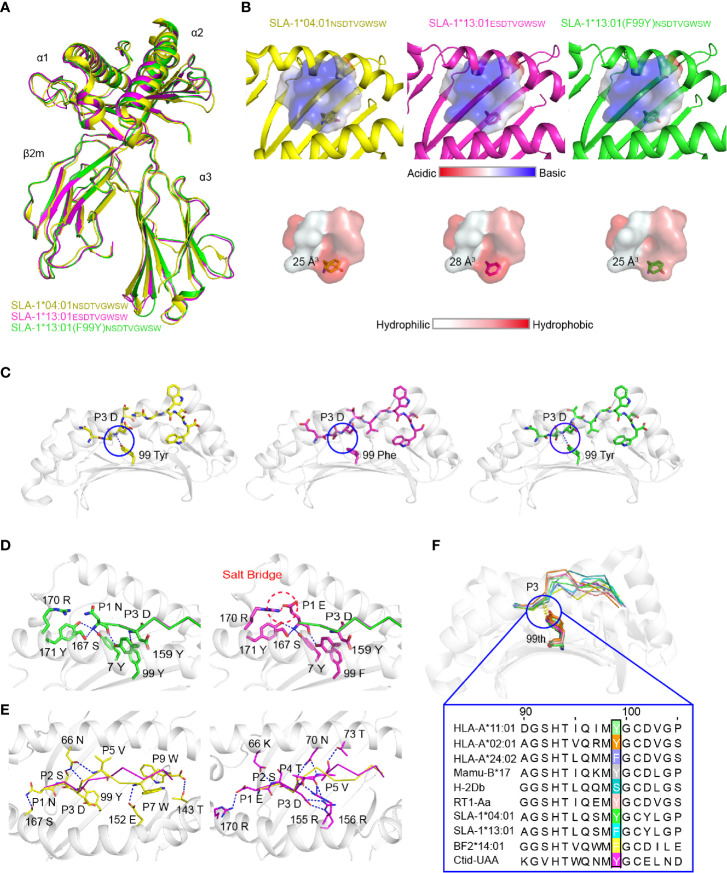
Structural analysis of pSLA-1 complexes. **(A)** The overall structural comparison between pSLA-1*04:01_NW9_ (yellow), pSLA-1*13:01_EW9_ (magenta), and pSLA-1*13:01 (F99Y)_NW9_ (green). The structures are presented in cartoon form. **(B)** Visualization of the surface charge and hydrophobicity of the ABG. The color indicates different properties according to the caliper at the bottom. The sizes of the pocket space volumes are labeled. **(C)** The key forces formed by peptides with residue 99 of the pSLA-1 complexes. The hydrogen bonds are indicated by blue dashed lines. **(D)** The forces between P1 and the pocket A were compared in the structures of SLA-1*13:01_EW9_ and SLA-1*13:01 (F99Y)_NW9_. Red dashed lines represent salt bridges formed by P1-Glu with 170^Arg^. Blue lines show hydrogen bonds between ABG and peptides. **(E)** Insight into the impact of extra forces on the peptide conformation from SLA-1*04:01_NW9_ and SLA-1*13:01_EW9_. **(F)** Structure-based sequence alignment of residue 99 of representative crystallized MHC class I molecules. The dashed lines indicate conserved hydrogen bonds with the P3 backbone.

To understand the mechanism by which micropolymorphism 99 affected peptide binding, two complexes, SLA-1*13:01 with peptide EW9 (pSLA-1*13:01_EW9_) and SLA-1*13:01 (F99Y) with peptide NW9 (pSLA-1*13:01 (F99Y)_NW9_), were crystallized. Both crystals were in the C121 space group with resolutions of 1.8 Å and 2.4 Å, respectively (**Table 2**). pSLA-1*13:01_EW9_ and pSLA-1*13:01 (F99Y)_NW9_ displayed a canonical MHC-I complex structure, which superposed well with the overall structure of pSLA-1*04:01 with peptide NW9 (pSLA-1*04:01_NW9_) that we resolved previously (PDB code 3QQ3) (**Figure 3A**). Residue 99 was involved in the D pocket (**Figure 1A**), but the structural analysis showed that the 99^Tyr/Phe^ micropolymorphism did not greatly alter the nature of the D pocket, regardless of the space volume, charge, and hydrophilic properties (**Figure 3B**).

**Figure 4:**
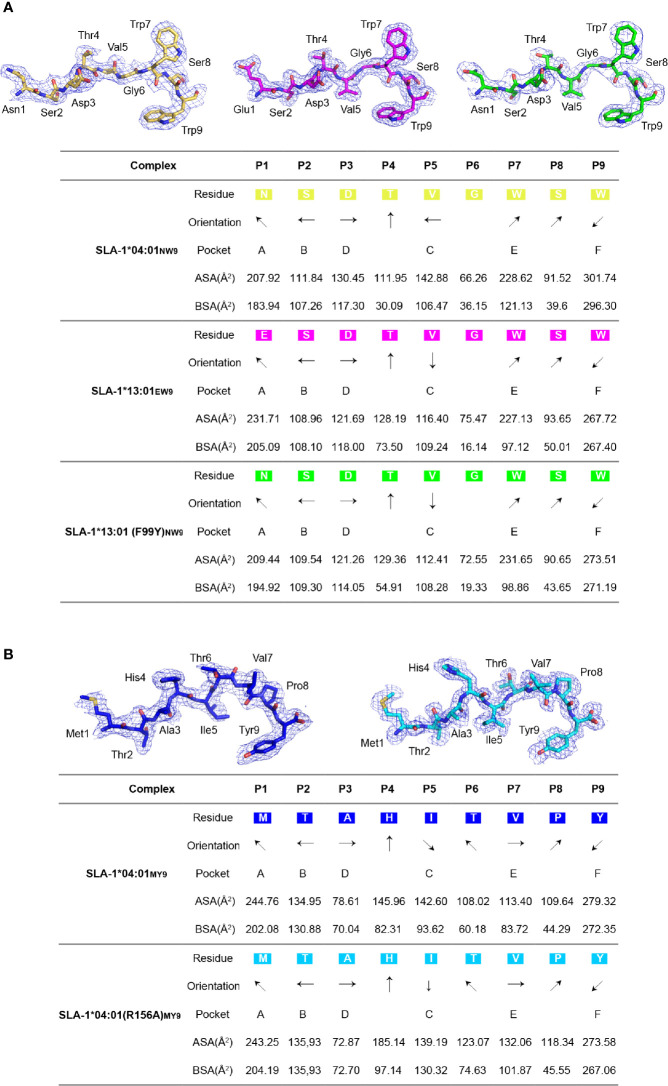
The electron density and overall conformation of the structurally defined peptides. Electron densities and overall conformations of peptides from the solved pSLA-1 complexes. Simulated CNS annealing omit maps calculated for the peptides are shown in blue at a contour of 1.0. General side chain orientations and the different interfacing areas of peptides presented in a table, as viewed in profile from the peptide N-terminus toward the C-terminus. Black arrows indicate the directions in which the residues point: up is toward the TCR, down is toward the floor of the ABG, left is toward the α1 helix domain, and right is toward the α2 helix domain. Pockets accommodating each residue are listed under the corresponding anchors within the ABG. ASA, accessible surface area of each residue; BSA, buried surface area of the residues. **(A)** The presentation of NW9 and EW9 peptides from pSLA-1*04:01_NW9_, pSLA-1*13:01_EW9_, and pSLA-1*13:01 (F99Y)_NW9_. **(B)** The presentation of the MY9 peptide from pSLA-1*04:01_MY9_ and pSLA-1*04:01 (R156A)_MY9_.

The comparison of the peptides revealed a significant difference that 99^Tyr^ could form a hydrogen bond with the carbon backbone of P3, but 99^Phe^ could not (**Figure 3C**). The clear electron density map indicated that the peptide conformations and interactions were stable and credible (**Figure 4A**). The hydrogen bond formed between 99^Tyr^, and the main chain of the P3 residue did not impact the interactions of the sidechain of the P3 residue and the D pocket but significantly enhanced the total binding affinity of NW9 (**Figures 2B, E**). Therefore, the microvariation of 99^Tyr/Phe^ could affect peptide binding but could not change the nature of the D pocket and the preference of the P3 residue.

To understand why EW9 can stabilize SLA-1*13:01 but not NW9, the force between P1 and the A pocket was analyzed (**Figure 3D**). In addition, some conservative backbone hydrogen bonds between residues in the A pocket and P1, such as 7^Tyr^, 167^Ser^, 171^Tyr^, and 170^Arg^, of SLA-1*13:01, formed a salt bridge with P1-Glu to stabilize the peptide (**Figure 3D**). The CD spectrum showed that the salt bridge between P1-Glu and 170^Arg^ almost compensated for the loss of the hydrogen bond between the backbone of the P3 residue and 99^Tyr^ (**Figure 2E**). Thus, the absence of the backbone hydrogen bond between 99^Phe^ and P3 residue should be compensated by other pockets to some extent, even if the affinity is not be the same as before.

The mutant and CD experiments indicated that the remaining four different amino acids between pSLA-1*04:01_NW9_ and pSLA-1*13:01_EW9_ did not play a major role in the peptide-binding affinity, but they were responsible for the peptide conformation variation. The peptide conformation of pSLA-1*13:01 was almost the same as that of pSLA-1*13:01 (F99Y) (RMSD = 0.175 Å), but it was quite different from that of pSLA-1*04:01 (RMSD = 1.110 Å). The two peptides of pSLA-1*13:01 and pSLA-1*13:01 (F99Y) had similar orientations, especially in P1, P2, P3, and PΩ residues, because these residues are anchored in pockets A, B, D, and F (**Figures 3D** and **Figure 4A**). Except for the conservative hydrogen bond between pSLA-1*04:01_NW9_ and pSLA-1*13:01_EW9_, 66^Asn^ and 152^Glu^ of pSLA-1*04:01_NW9_ provided horizontal tensions on both sides of the peptide (**Figure 3E**). In comparison, 66^Lys^,70^Asn^, 73^Thr^,155^Arg^, and 156^Arg^ from SLA-1*13:01_EW9_ were stabilized the peptide conformation and required additional hydrogen bonding force to pull down the peptide (**Figure 3E**).

We compared micropolymorphism 99 in other representative crystallized MHC-I molecules from the Immuno Polymorphism Database (IPD, https://www.ebi.ac.uk/ipd/) and PDB (http://www.rcsb.org/). In contrast to micropolymorphism 156, we found that the 99^Tyr^ micropolymorphism conserved in most species could form a backbone hydrogen bond with P3 of peptides (**Figure 3F**). In addition, 99^Tyr^ accounted for 80.6% (approximately 5734) of all HLA-A alleles, but 99^Phe^ was mainly concentrated in HLA-A*24 alleles, accounting for 14.5% of all HLA-A alleles (data not shown). Residue 99 was more polymorphic in HLA-C alleles than in HLA-A and HLA-B alleles. Other exceptions included, but were not limited to, mouse and chicken, as both alleles contained 99^Ser^, 99^Phe^ and 99^Tyr^ (**Figure 3F**). Although 99^Tyr^ played a dominant role in the evolution of the species, mutations at this residue greatly increased the restriction of epitopes. Therefore, 99^Tyr^ has been shown to favor multispecies MHC-I binding peptides for the first time, but its mutations are also significant for improving epitope restrictions.

### Immunopeptidomes Showed that the R156A Mutation Can Expand the Range of Peptide Binding of SLA-1*04:01 by Affecting the P3 Residues

**Figure 5:**
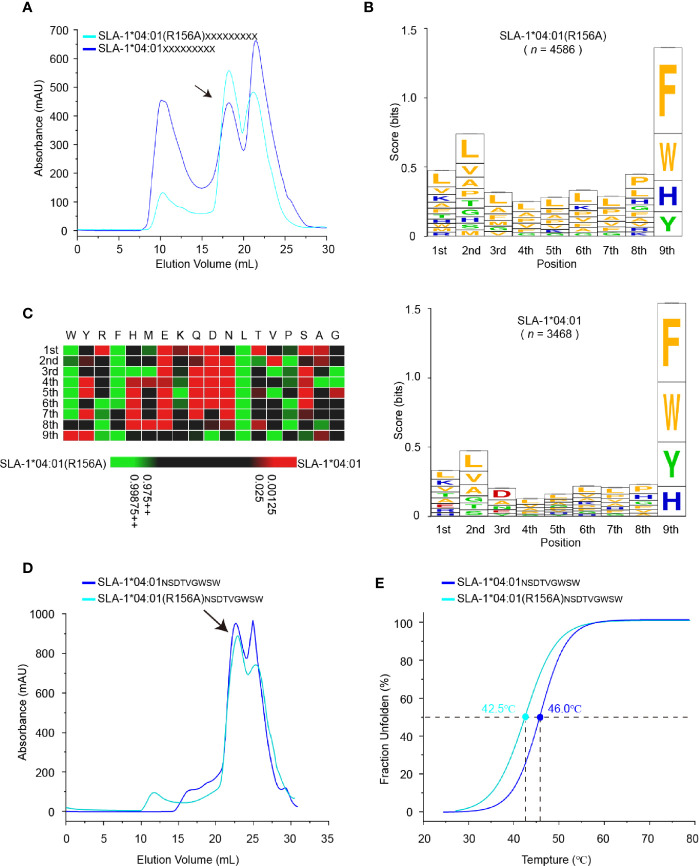
Determination of motif changes in SLA-1*04:01 caused by R156A. **(A)** Visual display of SLA-1*04:01 and its mutant SLA-1*04:01 (R156A) *in vitro* refolding efficiencies with random nonapeptide repertoire by gel filtration chromatograms. The black arrows point to the peak of the compound. **(B)** Visual analysis of the identified peptides by the WebLogo website (http://weblogo.berkeley.edu/). Amino acids are represented by their respective single-letter code with their heights scaled to prevalence and colors representing basic (blue), acidic (red), polar (green), and hydrophobic (orange) residues. Only amino acids with a 5% or greater prevalence are depicted. *n* is the number of peptides within the data set. Each column of amino acids has an error bar at the top. The height of the y-axis is the maximum entropy for the given sequence type (log_2_20 = 4.3 bits). **(C)** Comparison of motifs between alleles and their mutant *via* heatmap analysis on the IceLogo website (https://iomics.ugent.be/icelogoserver/). The color (green or red) indicates a significant difference (*P* < 0.05) in the amino acid at the position between the two allele motifs. **(D)** Visual display of SLA-1*04:01 and its mutant SLA-1*04:01 (R156A) *in vitro* refolding efficiencies with peptide NSDTVGWSW by gel filtration chromatograms. The black arrows point to the peak of the compound. **(E)** Thermal stabilities of pSLA-1*04:01_NW9_ and pSLA-1*04:01 (R156A)_NW9_ analyzed by the CD spectrum. The stabilities can be measured by the *Tm* value. The *Tm* values of the complexes are labeled.

To map R156A-dependent changes in the unbiased peptide-binding motif, SLA-1*04:01 and SLA-1*04:01 (R156A) were refolded with s*β*2-M and a random nonapeptide library *in vitro*, and the complex peaks were collected (**Figure 5A**). The eluted immunopeptidomes of SLA-1*04:01 and SLA-1*04:01 (R156A) were determined by MS *de novo* sequencing (34). The number of identified peptides from pSLA-1*04:01 (R156A) (*n* = 4586*)* was greater than that from pSLA-1*04:01 (*n* = 3468) (**Table S1**). This result was consistent with those shown in **Table 1** and our previous peptide-binding data (30).

**Figure 6:**
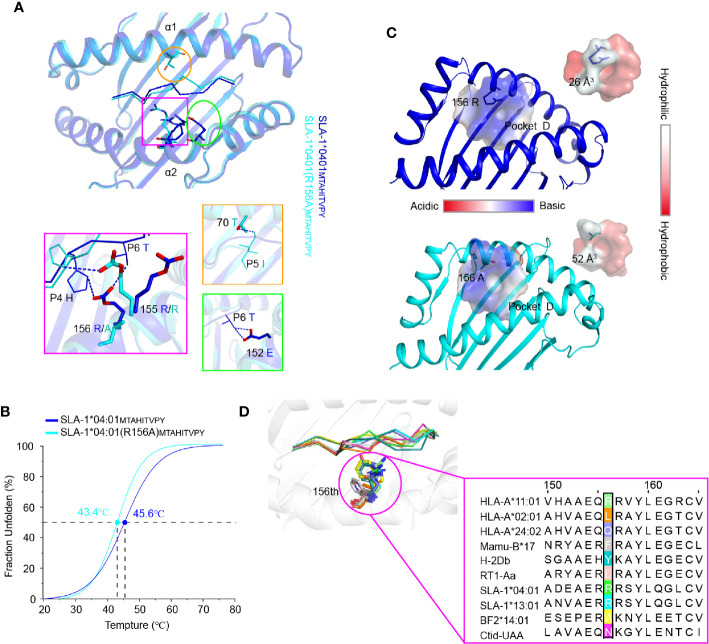
The structural basis of the residue 156 affecting peptide plasticity. **(A)** Comparison of the peptide conformation between pSLA-1*04:01_MY9_ (blue) and pSLA-1*04:01 (R156A)_MY9_ (cyans). The extra hydrogen bonding forces causing the conformational change are indicated by blue dashed lines. **(B)** Thermal stabilities of pSLA-1*04:01_MY9_ and pSLA-1*04:01 (R156A)_MY9_ analyzed by the CD spectrum. The stabilities can be measured by the *Tm* value. The *Tm* values of the complexes are labeled. **(C)** Character analysis of the D pocket. Residue 156 is shown in ball-and-stick form. D pockets are shown through the surface. The color indicates the surface charge and hydrophobicity, according to the caliper. The sizes of the pocket space volumes are labeled. **(D)** Structure-based sequence alignment of residue 156 of representative crystallized MHC class I molecules. Residue 156 is shown in ball-and-stick form.

The peptide-binding motif of SLA-1*04:01 (R156A) was almost the same as that of SLA-1*04:01, except for the significant difference in the P3 residues (*P* < 0.05) (**Figures 5B, C**). This result was reasonable because residue 156 is located in the D pocket, which accommodates the P3 residue of peptides. The R156A mutation preferred the P3 residue transition from acidic and small residues to hydrophobic residues (**Figure 5B**). Notably, SLA-1*04:01 (R156A) could still bind the P3-Asp peptides, such as peptide NW9, although they only account for 1.5% (**Figure 5D** and **Table S1**). This is because once Arg156 is mutated to Ala, it is equivalent to removing the strict restriction on peptide binding. The space of the D pocket became large enough to accommodate the side chains of P3 residues of different sizes and charged properties (**Figure 6C** and **Table S1**). But on the other hand, the mutation of R156A made the D pocket hydrophobic, so it preferred to bind hydrophobic P3 residues. To further confirm, we measured its stability by CD spectrum. The *Tm* value of the pSLA-1*04:01 (R156A)_NW9_ (*Tm* = 42.5°C) was significantly lower than that of the pSLA-1*04:01_NW9_ (*Tm* = 46.0°C), which meant that the R156A mutation lead to a decrease in the stability of its binding to the P3-D peptides (**Figure 5E**). Therefore, R156A mutation allowed pocket D to bind more types of P3 residues (including P3-Asp), but preferred hydrophobic amino acids, which also explained why SLA-1*04:01 (R156A) could bind to more peptides than SLA-1*04:01 (**Figure 5B** and **Table S1**).

### Structural Insights Into the Impact of the R156A Mutation on Peptide Binding by X-Ray Crystallography Structures and the CD Spectrum

To clearly understand the mechanism of the R156A mutation affecting peptide binding, the crystals of pSLA-1*04:01 and pSLA-1*04:01 (R156A) were both solved with the same peptide MTAHITVPY derived from FMDV (MY9 in short). The two structures, pSLA-1*04:01_MY9_ and pSLA-1*04:01 (R156A)_MY9_, were determined in the C121 and P12_1_1 space groups with resolutions of 2.0 Å and 1.8 Å, respectively (**Table 2**). The MY9 peptides in the two structures had a clear electronic density map and adopted the traditional overall “M” conformation (**Figure 4B**). However, a comparison of the main chain structure of the peptide (root-mean-square deviation (RMSD) = 1.172 Å) showed a significant difference in the central region of the P5, P6, and P7 residues, which are recognized by TCR (**Figure 6A**). In pSLA-1*04:01_MY9_, 156^Arg^ formed a hydrogen bond with P4-His, but in pSLA-1*04:01 (R156A)_MY9,_ 155^Arg^ interacted with P4-His (**Figure 6A**). This led to conformational changes in P4-His in the two structures (**Figure 6A**). In pSLA-1*04:01_MY9_, 156^Arg^ also bound to P6-Thr and pulled the sidechain of P6-Thr toward the *α*2 helix, causing 152^Glu^ to form two hydrogen bonds with P6-Thr (**Figure 6A**). In pSLA-1*04:01 (R156A)_MY9_, 70^Thr^ on the opposite side of the groove provided a hydrogen bond to the main chain of P5-Ile, resulting in the middle section of the MY9 peptide being pulled toward the *α*1 helix (**Figure 6A**). To determine the peptide binding stability, the two complexes were analyzed by the CD spectrum. The *Tm* value of pSLA-1*04:01 (*Tm* = 45.6°C) was slightly higher than that of pSLA-1*04:01(R156A) (*Tm* = 43.4°C) (**Figure 6B**). The CD results were consistent with the structural analysis, indicating that micropolymorphism 156 could alter the peptide binding stability (**Figures 6A, B**).

Residue 156 is part of the D pocket and can directly affect the pocket properties, such as the geometry, charge distribution and hydrophobicity. Compared with 156^Arg^, 156^Ala^ made the D pocket of SLA-1*04:01 (R156A) larger and more hydrophobic than that of SLA-1*04:01 (**Figure 6C**). The space volumes of the D pockets of SLA-1*04:01 and SLA-1*04:01 (R156A) were 26 and 52 Å^3^, respectively. This explained why SLA-1*04:01 (R156A) preferred to bind to hydrophobic P3 residues, regardless of the size of their sidechain (**Table 1** and **Figure 5B**). Moreover, 156^Ala^ did not change the negatively charged characteristics of the entire D pocket, probably because of the surrounding amino acids such as 155^Arg^, so SLA-1*04:01 (R156A) could still bind P3 acidic residues (**Table 1**). The change in the nature of the D pocket caused by the R156A mutation was the structural basis of SLA-1*04:01(R156A) binding more peptides than SLA-1*04:01. These results indicated that micropolymorphism 156 had the capacity to influence the plasticity of peptides and further TCR recognition.

Alignment of representative MHC-I molecules of different species revealed that residue 156 was one of the most highly variable residues (**Figure 6D**). The great variation of residue 156 helped shape the complex diversity of the D pocket and alter the peptide binding of MHC-I. Therefore, micropolymorphism 156 facilitated the alteration of epitope plasticity and benefited to the activation of various cytotoxic T lymphocyte (CTL) immune responses.

## Discussion

The high degree of micropolymorphisms enables MHC-I molecules to present a wide range of antigenic peptides and activate T-cell immune responses, and even a single residue mutation can drastically alter the peptide presentation of the MHC-I molecule and further affect disease resistance (18, 20–22, 25). Numerous alleles and amino acid variations make it difficult to fully illustrate how micropolymorphism alter the structure and function of MHC-I molecules. Recently, LC-MS/MS was widely used to survey the eluted immunopeptidome of MHC-I (28), and the influence of the HLA-I micropolymorphism could be determined by an improved method combining LC-MS/MS and CRISPR technology (2, 29). However, the current MS method is a data-dependent acquisition method and is difficult to use for the numerous animal MHC-I alleles lacking essential study conditions. We have established an *in vitro* method RPLD-MS using a random peptide library that combines LC-MS/MS and *de novo* sequencing to identify the peptide-binding motif of MHC-I (34), which is a data-independent acquisition MS method and is suitable to overcome limitations such as the absence of certain antibodies and cell lines. The results of the SLA-1*0401-bound peptidome determined by RPLD-MS were very reproducible. We measured the peptidome eluted from SLA-1*04:01 molecule twice, and collected peptides with a score of more than 50 in the *de novo* MS results for statistical analysis. The two results were not significantly different, and peptide-binding motif of SLA-1*04:01 determined by RPLD-MS perfectly matched our previous data and showed a more complete landscape (30), supporting the accuracy and comprehensiveness of this method. The method was also sensitive enough to reflect how the R156A mutation and 99^Tyr/Phe^ variation influenced the binding of peptides to SLA-1*04:01, SLA-1*04:01(R156A), and SLA-1*13:01. Compared with existing *in/ex vivo* MS methods, this *in vitro* method could not reflect the process of intracellular peptide processing, but it also avoided the influence of intracellular factors on the peptide presentation of MHC-I, focusing only on the impact of the micropolymorphism. As an *in vitro* test method, it could be directly used for a single MHC-I allele without cell lines and antibodies. Moreover, the low cost, simple procedure, short cycle time, and credibility make RPLD-MS very suitable for studying numerous and highly polymorphic unknown MHC-I alleles, as confirmed by the peptidome sequencing and structural analysis of the MHC-I molecules of bats and *Xenopus laevis* (34, 35). We believe that the impact of the MHC-I micropolymorphism will be better studied by the combination of this *in vitro* data-independent acquisition MS method and current *in/ex vivo* data-dependent acquisition MS methods.

**Figure 7:**
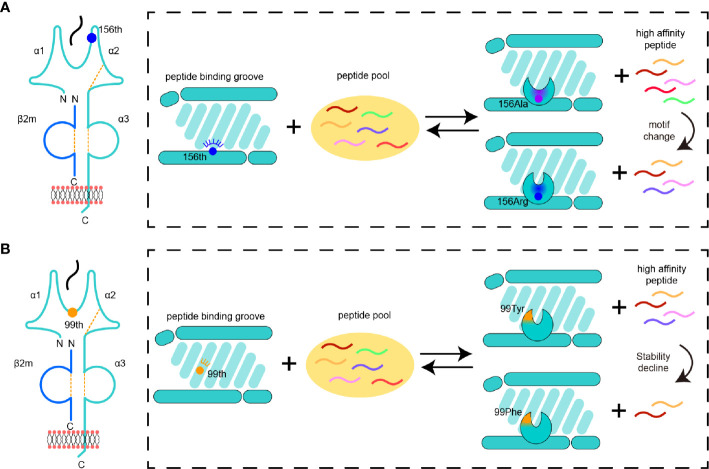
Pattern diagram of micropolymorphism affecting MHC-I peptide presentation. **(A)** The impacts of mutant 156 on the D pocket properties and peptide binding of MHC-I. The color of a peptide indicates a set of peptides with a specific motif. The change in the pocket properties is represented by different colors. **(B)** The impacts of variation 99 on the MHC-I D pocket properties and peptide binding.

In this study, we found two distinguished manners by which the microploymorphism affects the peptide binding of MHC-I (**Figure 7**). The first manner has always been a concern, that is, the key amino acid changes the nature of the pocket, resulting in a change in the preference of amino acids accommodated in the pocket (**Figure 7A**). The impacts of micropolymorphism at residue 156 on peptide presentation and CTL immunity were found during comparative studies of HLA-B*35:01/08 (156^Leu/Arg^) (19, 52) and HAL-B*44:02/03 (156^Asp/Leu^) (23, 53, 54). The 156^Arg^ in HLA-B*35:08 was shown to prefer P5 E/D as the secondary anchor residues outside the primary peptide anchor pockets (B and F pocket) (23). In SLA-I molecules, residue 156 also plays a critical role in fixing peptides (30, 50, 55). The positively charged 156^Arg^ made the D pocket of SLA-1*04:01 prefer negatively charged P3 residues. We then found that the R156A mutation could expand the scope of peptide binding, but at that time we could not provide a clear overall description of this expansion. We can now easily study the alteration of the anchor residues of SLA-1*0401 caused by R156A mutation using RPLD-MS. The structural analysis and peptidomes determined by RPLD-MS showed that once 156^Arg^ was mutated into Ala, the nature of the D pocket was altered, becoming larger and more hydrophobic. These changes made the D pocket of SLA-1*04:01 (R156A) accommodate more types of residues but show a preference for hydrophobic P3 residues (**Figure 7A**).

The second manner was unexpected; the residue interacted with the main chain of the peptide, and even if it did not change the nature of the pocket greatly, it still played an important role in peptide binding (**Figure 7B**). Although the micropolymorphism of residue 99 has been found in HLA-A*02:01 and HLA-A*02:07 (99^Tyr/Cys^), and this mutation is able to affect the conformation and affinity of peptide binding, potentially even Epstein–Barr virus infection, its alteration of the peptide binding motif is still not clear (56, 57). Here we found five different residues in SLA-1*04:01 and SLA-1*13:01, but only the 99^Tyr/Phe^ mutant was verified as the key to causing the differences in binding peptides between the two SLA-I allomorphs (**Figure 7B**). Residue 99 was located on the bottom edge of the D pocket, and 99^Tyr^ could form a hydrogen bond with the main carbon chain of the P3 residue. The D pocket was not altered greatly by 99^Tyr/Phe^, so its preference for P3 residues persisted. The CD spectrum verified that this hydrogen bond could dramatically influence the stability of the MHC class I complex. The lack of this hydrogen bond in SLA-1*13:01 caused by 99^Phe^ led to a sharp decline in the peptide-binding affinity. To compensate for this loss, extra complementary binding forces were needed. This compensation was also not reflected in the traditional key anchoring pockets such as P2 and P9 because these residues were already the most suitable for pocket accommodation. Therefore, other pockets must provide additional binding to maintain peptide binding, such as the salt bridge between the P1 residue and Arg^170^ in the A pocket. Thus, among the peptides bound by SLA-1*04:01, only some of the high affinity peptides could interact with SLA-1*13:01. As reflected in the immunopeptideomics results, SLA-1*13:01 bound fewer peptides than SLA-1*04:01, and there were no changes in anchor residues at P2, P3, and P9, but the preference for P1 residues appeared variable. The mechanism identified herein helps to reveal why the micropolymorphism of residue 99 has an important impact on the peptide presentation of HLA-I molecules (58).

In summary, using a newly developed data-independent acquisition MS method, we present a comprehensive and in-depth description of how micropolymorphisms 156 and 99 alter the peptide presenting plasticity of SLA-I in two different ways. Insight into the data could lay the foundation for epitope prediction and vaccine development.

## Data Availability Statement

The datasets presented in this study can be found in online repositories. The names of the repository/repositories and accession number(s) can be found in the article/**Supplementary Material**.

## Author Contributions

NZ, CX, and XW conceived and designed the study. XW and SW performed the experiments. ZLL, ZBL, and SQW collected and analyzed the crystal data. ZQ, BZ, and RL analyzed the LC-MS/MS data. XW and NZ wrote the manuscript. NZ and CX revised the manuscript. All authors contributed to the article and approved the submitted version.

## Funding

This work was supported financially by grants from the National Natural Science Foundation of China (NSFC) (31201887, http://www.nsfc.gov.cn) and the Natural Science Foundation of Beijing Municipality (6182029, http://www.bjkw.gov.cn/).

## Conflict of Interest

The authors declare that the research was conducted in the absence of any commercial or financial relationships that could be construed as a potential conflict of interest.

## Abbreviations

MHC-I, major histocompatibility complex class I; β2m, β-2-microglobulin; ABG, antigen binding groove; MS, mass spectrometry; LC-MS/MS, liquid chromatography-tandem mass spectrometry; RPLD-MS, random peptide library combined with mass spectrometry (MS) *de novo* sequencing; RMSD, root-mean-square deviation; *Tm*, midpoint transition temperature; ASA, accessible surface area; BSA, buried surface area.

## References

[1] Kaufman J. Unfinished Business: Evolution of the MHC and the Adaptive Immune System of Jawed Vertebrates. Annu Rev Immunol (2018) 36:383–409. 10.1146/annurev-immunol-051116-052450 29677478

[2] Illing PT Pymm P Croft NP Hilton HG Jojic V Han AS. HLA-B57 micropolymorphism defines the sequence and conformational breadth of the immunopeptidome. Nat Commun (2018) 9:4693. 10.1038/s41467-018-07109-w 30410026 PMC6224591

[3] Garrett TP Saper MA Bjorkman PJ Strominger JL Wiley DC. Specificity pockets for the side chains of peptide antigens in HLA-Aw68. Nature (1989) 342:692–6. 10.1038/342692a0 2594067

[4] Madden DR. The three-dimensional structure of peptide-MHC complexes. Annu Rev Immunol (1995) 13:587–622. 10.1146/annurev.iy.13.040195.003103 7612235

[5] Reche PA Reinherz EL. Sequence variability analysis of human class I and class II MHC molecules: functional and structural correlates of amino acid polymorphisms. J Mol Biol (2003) 331:623–41. 10.1016/s0022-2836(03)00750-2 12899833

[6] Adams EJ Luoma AM. The adaptable major histocompatibility complex (MHC) fold: structure and function of nonclassical and MHC class I-like molecules. Annu Rev Immunol (2013) 31:529–61. 10.1146/annurev-immunol-032712-095912 23298204

[7] Olson E Geng J Raghavan M. Polymorphisms of HLA-B: influences on assembly and immunity. Curr Opin Immunol (2020) 64:137–45. 10.1016/j.coi.2020.05.008 PMC7772265 32619904

[8] Sidney J Peters B Frahm N Brander C Sette A. HLA class I supertypes: a revised and updated classification. BMC Immunol (2008) 9:1. 10.1186/1471-2172-9-1 18211710 PMC2245908

[9] Uebel S Kraas W Kienle S Wiesmuller KH Jung G Tampe R. Recognition principle of the TAP transporter disclosed by combinatorial peptide libraries. Proc Natl Acad Sci USA (1997) 94:8976–81. 10.1073/pnas.94.17.8976 PMC22991 9256420

[10] Purcell AW Gorman JJ Garcia-Peydro M Paradela A Burrows SR Talbo GH. Quantitative and qualitative influences of tapasin on the class I peptide repertoire. J Immunol (2001) 166:1016–27. 10.4049/jimmunol.166.2.1016 11145681

[11] Williams AP Peh CA Purcell AW McCluskey J Elliott T. Optimization of the MHC class I peptide cargo is dependent on tapasin. Immunity (2002) 16:509–20. 10.1016/s1074-7613(02)00304-7 11970875

[12] Zernich D Purcell AW Macdonald WA Kjer-Nielsen L Ely LK Laham N. Natural HLA class I polymorphism controls the pathway of antigen presentation and susceptibility to viral evasion. J Exp Med (2004) 200:13–24. 10.1084/jem.20031680 15226359 PMC2213310

[13] Rizvi SM Salam N Geng J Qi Y Bream JH Duggal P. Distinct assembly profiles of HLA-B molecules. J Immunol (2014) 192:4967–76. 10.4049/jimmunol.1301670 PMC4117407 24790147

[14] Wieczorek M Abualrous ET Sticht J Alvaro-Benito M Stolzenberg S Noe F. Major Histocompatibility Complex (MHC) Class I and MHC Class II Proteins: Conformational Plasticity in Antigen Presentation. Front Immunol (2017) 8:292:292. 10.3389/fimmu.2017.00292 28367149 PMC5355494

[15] Matzaraki V Kumar V Wijmenga C Zhernakova A. The MHC locus and genetic susceptibility to autoimmune and infectious diseases. Genome Biol (2017) 18:76. 10.1186/s13059-017-1207-1 28449694 PMC5406920

[16] Naranbhai V Carrington M. Host genetic variation and HIV disease: from mapping to mechanism. Immunogenetics (2017) 69:489–98. 10.1007/s00251-017-1000-z PMC5537324 28695282

[17] McAulay KA Jarrett RF. Human leukocyte antigens and genetic susceptibility to lymphoma. Tissue Antigens (2015) 86:98–113. 10.1111/tan.12604 26189878

[18] Macdonald WA Chen Z Gras S Archbold JK Tynan FE Clements CS. T cell allorecognition via molecular mimicry. Immunity (2009) 31:897–908. 10.1016/j.immuni.2009.09.025 20064448

[19] Macdonald WA Purcell AW Mifsud NA Ely LK Williams DS Chang L. A naturally selected dimorphism within the HLA-B44 supertype alters class I structure, peptide repertoire, and T cell recognition. J Exp Med (2003) 198:679–91. 10.1084/jem.20030066 PMC2194191 12939341

[20] Bowness P. HLA-B27. Annu Rev Immunol (2015) 33:29–48. 10.1146/annurev-immunol-032414-112110 25861975

[21] Schittenhelm RB Sian TC Wilmann PG Dudek NL Purcell AW. Revisiting the arthritogenic peptide theory: quantitative not qualitative changes in the peptide repertoire of HLA-B27 allotypes. Arthritis Rheumatol (Hoboken NJ) (2015) 67:702–13. 10.1002/art.38963 25418920

[22] Schittenhelm RB Sivaneswaran S Lim Kam Sian TC Croft NP Purcell AW. Human Leukocyte Antigen (HLA) B27 Allotype-Specific Binding and Candidate Arthritogenic Peptides Revealed through Heuristic Clustering of Data-independent Acquisition Mass Spectrometry (DIA-MS) Data. Mol Cell Proteomics MCP (2016) 15:1867–76. 10.1074/mcp.M115.056358 PMC5083097 26929215

[23] Burrows JM Wynn KK Tynan FE Archbold J Miles JJ Bell MJ. The impact of HLA-B micropolymorphism outside primary peptide anchor pockets on the CTL response to CMV. Eur J Immunol (2007) 37:946–53. 10.1002/eji.200636588 17357107

[24] Tynan FE Reid HH Kjer-Nielsen L Miles JJ Wilce MC Kostenko L. A T cell receptor flattens a bulged antigenic peptide presented by a major histocompatibility complex class I molecule. Nat Immunol (2007) 8:268–76. 10.1038/ni1432 17259989

[25] Kloverpris HN Cole DK Fuller A Carlson J Beck K Schauenburg AJ. A molecular switch in immunodominant HIV-1-specific CD8 T-cell epitopes shapes differential HLA-restricted escape. Retrovirology (2015) 12:20. 10.1186/s12977-015-0149-5 25808313 PMC4347545

[26] Bossi G Mannarino S Pietrogrande MC Salice P Dellepiane RM Cremaschi AL. Genetic epistasis between killer immunoglobulin-like receptors and human leukocyte antigens in Kawasaki disease susceptibility. Genes Immun (2015) 16:481–7. 10.1038/gene.2015.34 26335810

[27] Alvarez B Barra C Nielsen M Andreatta M. Computational Tools for the Identification and Interpretation of Sequence Motifs in Immunopeptidomes. Proteomics (2018) 18:e1700252. 10.1002/pmic.201700252 29327813 PMC6279437

[28] Gfeller D Bassani-Sternberg M. Predicting Antigen Presentation-What Could We Learn From a Million Peptides? Front Immunol (2018) 9:1716. 10.3389/fimmu.2018.01716 30090105 PMC6068240

[29] Abelin JG Keskin DB Sarkizova S Hartigan CR Zhang W Sidney J. Mass Spectrometry Profiling of HLA-Associated Peptidomes in Mono-allelic Cells Enables More Accurate Epitope Prediction. Immunity (2017) 46:315–26. 10.1016/j.immuni.2017.02.007 PMC5405381 28228285

[30] Zhang N Qi J Feng S Gao F Liu J Pan X. Crystal structure of swine major histocompatibility complex class I SLA-1 0401 and identification of 2009 pandemic swine-origin influenza A H1N1 virus cytotoxic T lymphocyte epitope peptides. J Virol (2011) 85:11709–24. 10.1128/JVI.05040-11 PMC3209268 21900158

[31] Gao C He X Quan J Jiang Q Lin H Chen H. Specificity Characterization of SLA Class I Molecules Binding to Swine-Origin Viral Cytotoxic T Lymphocyte Epitope Peptides in Vitro. Front Microbiol (2017) 8:2524. 10.3389/fmicb.2017.02524 29326671 PMC5741678

[32] Tran NH Qiao R Xin L Chen X Liu C Zhang X. Deep learning enables de novo peptide sequencing from data-independent-acquisition mass spectrometry. Nat Methods (2019) 16:63–6. 10.1038/s41592-018-0260-3 30573815

[33] Tran NH Zhang X Xin L Shan B Li M. De novo peptide sequencing by deep learning. Proc Natl Acad Sci USA (2017) 114:8247–52. 10.1073/pnas.1705691114 PMC5547637 28720701

[34] Qu Z Li Z Ma L Wei X Zhang L Liang R. Structure and Peptidome of the Bat MHC Class I Molecule Reveal a Novel Mechanism Leading to High-Affinity Peptide Binding. J Immunol (2019) 202:3493–506. 10.4049/jimmunol.1900001 PMC6545463 31076531

[35] Ma L Zhang N Qu Z Liang R Zhang L Zhang B. A Glimpse of the Peptide Profile Presentation by Xenopus laevis MHC Class I: Crystal Structure of pXela-UAA Reveals a Distinct Peptide-Binding Groove. J Immunol (2020) 204:147–58. 10.4049/jimmunol.1900865 PMC6926391 31776204

[36] Chi H Chen H He K Wu L Yang B Sun RX. pNovo+: de novo peptide sequencing using complementary HCD and ETD tandem mass spectra. J Proteome Res (2013) 12:615–25. 10.1021/pr3006843 23272783

[37] Muth T Weilnbock L Rapp E Huber CG Martens L Vaudel M. DeNovoGUI: an open source graphical user interface for de novo sequencing of tandem mass spectra. J Proteome Res (2014) 13:1143–6. 10.1021/pr4008078 PMC3923451 24295440

[38] Caron E Kowalewski DJ Chiek Koh C Sturm T Schuster H Aebersold R. Analysis of Major Histocompatibility Complex (MHC) Immunopeptidomes Using Mass Spectrometry. Mol Cell Proteomics MCP (2015) 14:3105–17. 10.1074/mcp.M115.052431 PMC4762616 26628741

[39] Stormo GD Schneider TD Gold L Ehrenfeucht A. Use of the ‘Perceptron’ algorithm to distinguish translational initiation sites in E. coli. Nucleic Acids Res (1982) 10:2997–3011. 10.1093/nar/10.9.2997 7048259 PMC320670

[40] Jones DT. Protein secondary structure prediction based on position-specific scoring matrices. J Mol Biol (1999) 292:195–202. 10.1006/jmbi.1999.3091 10493868

[41] Crooks GE Hon G Chandonia JM Brenner SE. WebLogo: a sequence logo generator. Genome Res (2004) 14:1188–90. 10.1101/gr.849004 PMC419797 15173120

[42] Colaert N Helsens K Martens L Vandekerckhove J Gevaert K. Improved visualization of protein consensus sequences by iceLogo. Nat Methods (2009) 6:786–7. 10.1038/nmeth1109-786 19876014

[43] Otwinowski Z Minor W. Processing of X-ray diffraction data collected in oscillation mode. Methods Enzymol (1997) 276:307–26. 10.1016/S0076-6879(97)76066-X 27754618

[44] McCoy AJ. Solving structures of protein complexes by molecular replacement with Phaser. Acta Crystallogr D Biol Crystallogr (2007) 63:32–41. 10.1107/s0907444906045975 17164524 PMC2483468

[45] Emsley P Lohkamp B Scott WG Cowtan K. Features and development of Coot. Acta Crystallogr D Biol Crystallogr (2010) 66:486–501. 10.1107/S0907444910007493 20383002 PMC2852313

[46] Murshudov GN Skubak P Lebedev AA Pannu NS Steiner RA Nicholls RA. REFMAC5 for the refinement of macromolecular crystal structures. Acta Crystallogr D Biol Crystallogr (2011) 67:355–67. 10.1107/S0907444911001314 PMC3069751 21460454

[47] Adams PD Afonine PV Bunkoczi G Chen VB Davis IW Echols N. PHENIX: a comprehensive Python-based system for macromolecular structure solution. Acta Crystallogr D Biol Crystallogr (2010) 66:213–21. 10.1107/s0907444909052925 PMC2815670 20124702

[48] Laskowski RA Moss DS Thornton JM. Main-chain bond lengths and bond angles in protein structures. J Mol Biol (1993) 231:1049–67. 10.1006/jmbi.1993.1351 8515464

[49] Fan S Wang Y Wang S Wang X Wu Y Li Z. Polymorphism and peptide-binding specificities of porcine major histocompatibility complex (MHC) class I molecules. Mol Immunol (2018) 93:236–45. 10.1016/j.molimm.2017.06.024 28751109

[50] Fan S Wu Y Wang S Wang Z Jiang B Liu Y. Structural and Biochemical Analyses of Swine Major Histocompatibility Complex Class I Complexes and Prediction of the Epitope Map of Important Influenza A Virus Strains. J Virol (2016) 90:6625–41. 10.1128/JVI.00119-16 PMC4944273 27170754

[51] Liang R Sun Y Liu Y Wang J Wu Y Li Z. Major Histocompatibility Complex Class I (FLA-E*01801) Molecular Structure in Domestic Cats Demonstrates Species-Specific Characteristics in Presenting Viral Antigen Peptides. J Virol (2018) 92(6):e01631–17. 10.1128/JVI.01631-17 29263258 PMC5827386

[52] Herman J Jongeneel V Kuznetsov D Coulie PG. Differences in the recognition by CTL of peptides presented by the HLA-B*4402 and the HLA-B*4403 molecules which differ by a single amino acid. Tissue Antigens (1999) 53:111–21. 10.1034/j.1399-0039.1999.530201.x 10090611

[53] Abels WC Manandhar T Kunze-Schumacher H Blasczyk R Bade-Doding C. The polymorphism at residue 156 determines the HLA-B*35 restricted peptide repertoire during HCMV infection. Immunogenetics (2018) 70:639–46. 10.1007/s00251-018-1077-z PMC6182399 30128813

[54] Manandhar T Kunze-Schumacher H Huyton T Celik AA Blasczyk R Bade-Doeding C. Understanding the obstacle of incompatibility at residue 156 within HLA-B*35 subtypes. Immunogenetics (2016) 68:247–60. 10.1007/s00251-015-0896-4 PMC4799800 26758079

[55] Pan X Zhang N Wei X Jiang Y Chen R Li Q. Illumination of PRRSV Cytotoxic T Lymphocyte Epitopes by the Three-Dimensional Structure and Peptidome of Swine Lymphocyte Antigen Class I (SLA-I). Front Immunol (2019) 10:2995. 10.3389/fimmu.2019.02995 31969884 PMC6960135

[56] Liu J Chen KY Ren EC. Structural insights into the binding of hepatitis B virus core peptide to HLA-A2 alleles: towards designing better vaccines. Eur J Immunol (2011) 41:2097–106. 10.1002/eji.201041370 21538979

[57] Huang X Hepkema B Nolte I Kushekhar K Jongsma T Veenstra R. HLA-A*02:07 is a protective allele for EBV negative and a susceptibility allele for EBV positive classical Hodgkin lymphoma in China. PloS One (2012) 7:e31865. 10.1371/journal.pone.0031865 22355400 PMC3280205

[58] van Deutekom HW Keşmir C. Zooming into the binding groove of HLA molecules: which positions and which substitutions change peptide binding most? Immunogenetics (2015) 67:425–36. 10.1007/s00251-015-0849-y PMC4498290 26040913

